# Non‐invasive hemodynamic profile of early COVID‐19 infection

**DOI:** 10.14814/phy2.14628

**Published:** 2020-10-28

**Authors:** Mattia Busana, Marco Schiavone, Antonio Lanfranchi, Giovanni Battista Forleo, Elisa Ceriani, Chiara Beatrice Cogliati, Alessio Gasperetti

**Affiliations:** ^1^ Department of Anesthesiology University Medical Center Göttingen Göttingen Germany; ^2^ Department of Cardiology ASST‐Fatebenefratelli Sacco Luigi Sacco Hospital University of Milan Milan Italy; ^3^ Department of Internal Medicine ASST‐Fatebenefratelli Sacco Luigi Sacco Hospital University of Milan Milan Italy

**Keywords:** acute respiratory failure, COVID‐19, echocardiography, hemodynamics, hyperdynamic state, hypoxemia

## Abstract

**Introduction:**

Little is known about the systemic and pulmonary macrohemodynamics in early COVID‐19 infection. Echocardiography may provide useful insights into COVID‐19 physiopathology.

**Methods:**

Twenty‐three COVID‐19 patients were enrolled in a medical ward. Gas exchange, transthoracic echocardiographic, and hemodynamic variables were collected.

**Results:**

Mean age was 57 ± 17 years. The patients were hypoxemic (PaO_2_/FiO_2_ = 273.0 ± 102.6 mmHg) and mildly hypocapnic (PaCO_2_ = 36.2 ± 6.3 mmHg, pH = 7.45 ± 0.03). Mean arterial pressure was decreased (86.7 [80.0–88.3] mmHg). Cardiac index was elevated (4.32 ± 0.90 L∙min^‐1^∙m^‐2^) and the resulting systemic vascular resistance index low (1,458 [1358–1664] dyn∙s∙cm^‐5^∙m^‐2^). The right heart was morphologically and functionally normal, with pulmonary artery pressure (PAPm, 18.0 ± 2.9 mmHg) and Total Pulmonary Resistances (TPR, 2.3 [2.1–2.7] mmHg∙l^‐1^∙min^‐1^) within normal limits. When stratifying for SVRI, patients with an SVRI value below the cohort median had also more severe oxygenation impairment and lower TPR, despite a similar degree of CXR infiltrates. Oxygen delivery index in this group resulted supranormal.

**Conclusions:**

In the early stages of COVID‐19 infection the hemodynamic profile is characterized by a hyperdynamic circulatory state with high CI and low SVRI, while the right heart is functionally unaffected. Our findings suggest that hypoxemia, viral sepsis or peripheral shunting are possible mechanisms for the vasodilation that dominates at this stage of the disease and may itself worsen the gas exchange.

## Graphical abstract

Early COVID‐19 infection is characterized by a hyperdynamic circulatory state, with low systemic resistances. Moreover, pulmonary pressures and pulmonary resistances are low. This suggests two main findings: at least in the initial stage, pulmonary vasodilation prevails over vasoconstriction and the hyperdynamic state may worsen the severe ventilation‐perfusion mismatch.

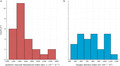

## 1 INTRODUCTION

COVID‐19 is a new disease and many of its physiopathological features are still unknown. Upon admission at the emergency department, patients often present with severe hypoxemia, sometimes despite relatively mild chest infiltrates. Indeed, some observations showed preserved lung mechanics despite high venous admixture (Gattinoni et al., 2020). This supports the hypothesis that the observed oxygenation impairment could, at least in part, depend also on severe ventilation‐perfusion (V_A_/Q) mismatch, rather than on true right‐to‐left shunt only. It is by now clear that COVID‐19 infection is whole‐body disease: together with severe pneumonia, hypercoagulability (Panigada et al., 2020), myocardial (Shi et al., 2020) and encephalic involvement (Mao et al., 2020), the presence of the virus has been detected in the endothelium of different organs (Varga et al., 2020). Moreover, autoptic findings showed a remarkable degree of intrapulmonary microthrombosis (Patel et al., 2020; Wichmann et al., 2020) and reports highlight the high incidence of overt pulmonary embolism (Patel et al., 2020; Poissy et al., 2020), which may also justify the high V_A_/Q mismatch observed (Tsang et al., 2005). Failure of the hypoxic vasoconstriction has been put forward as an alternative or concurrent mechanism for the development of low V_A_/Q regions (Gattinoni et al., 2020). Nevertheless, while this should lead to normal or even decreased pulmonary vascular resistances, widespread microthrombosis should have opposite effects on the pulmonary circulation with possible impairment of the right heart function. Little is known about the hemodynamic asset of COVID‐19 in the early stages of the disease and, unfortunately, the direct right heart catheterization out of an Intensive Care Unit does not appear feasible, especially during pandemics. Transthoracic echocardiography (TTE) is a non‐invasive alternative for a thorough hemodynamic evaluation, also feasible in the non‐critical patient. This study provides data that could help to understand the interplay between the macrohemodynamics and the mechanisms of the severe hypoxemia in the early stages of COVID‐19 infection.

## 2 MATERIALS AND METHODS

The study was approved by the ethical committee of the institution. Consecutive, non‐critical, non‐intubated COVID‐19 positive patients undergoing TTE from 22nd March to 30th of April were screened for enrollment in a medical ward. Patients with a history of ischemic, myocardial, valvular heart diseases, or suspicion of present acute cardiac illness were excluded. The arterial blood gas analysis was acquired either in room air or during the oxygen administration through low‐flow nasal cannulas or Venturi mask. The Inspired oxygen fraction (FiO_2_) was estimated accordingly. Continuous Positive Airway Pressure (CPAP) or Non‐Invasive Ventilation (NIV) were, at this stage, not applied. The Alveolar‐arterial O_2_ gradient (A‐aO_2_) was calculated as:

$$A‐{\mathrm{aO}}_{2}=\left({\mathrm{FiO}}_{2}∙713‐\frac{{\mathrm{PaCO}}_{2}}{0.8}\right)‐{\mathrm{PaO}}_{2}$$

The systemic blood pressures (systolic, diastolic, and mean) were measured non‐invasively.

TTE measurements and cardiac function analysis were performed in accordance with the American Society of Echocardiography recommendations. Stroke volume (SV) was calculated from the velocity‐time integral profile of PW Doppler on the left ventricular outflow tract (LVOT) and multiplied by the heart rate to calculate the cardiac output (CO). Systolic pulmonary artery pressure (PAPs) was assessed measuring the tricuspid regurgitation velocity and the right atrial pressure (RAP) was estimated through the collapsibility of the inferior vena cava (Rudski et al., 2010). Mean PAP (PAPm) was calculated as proposed by Chemla et al. (2004). The systemic vascular resistances (SVR) were calculated as:

$$\mathrm{SVR}=\frac{\mathrm{MAP}‐\mathrm{RAP}}{\mathrm{CO}}$$

SV and CO were divided by the Body Surface Area (BSA) to calculate the Stroke Index (SVI) and Cardiac Index (CI). The systemic vascular resistance index was calculated as:

$$\mathrm{SVRI}=\frac{\mathrm{MAP}‐\mathrm{RAP}}{\mathrm{CI}}$$

The Total Pulmonary Resistances (TPR) were calculated as PAPm/CO. The oxygen delivery (DO_2_) and the oxygen delivery index (DO_2_i) were calculated as the arterial oxygen content (CaO_2_) multiplied by CO and CI, respectively. The chest x‐ray scans (CXR) were were graded into five levels of severity according to Taylor et al (Taylor et al., 2015): normal = grade 1; patchy infiltrates/hyperinflation/bronchial wall thickening = grade 2; focal consolidation in no more than one lobe = grade 3; multifocal consolidation = grade 4; and diffuse alveolar consolidation = grade 5.

The normal distribution of the variables was assessed with the Shapiro–Wilk test. Data are presented as mean ± standard deviation or median [interquartile range] as appropriate. The statistical significance of the difference between the variables was assessed with Student's *t* test or Kruskal–Wallis test as appropriate. Two‐tailed *p* values <.05 were considered statistically significant. The data analysis was performed with Python 3.7.

## 3 RESULTS

### 3.1 Population

**Figure 1:**
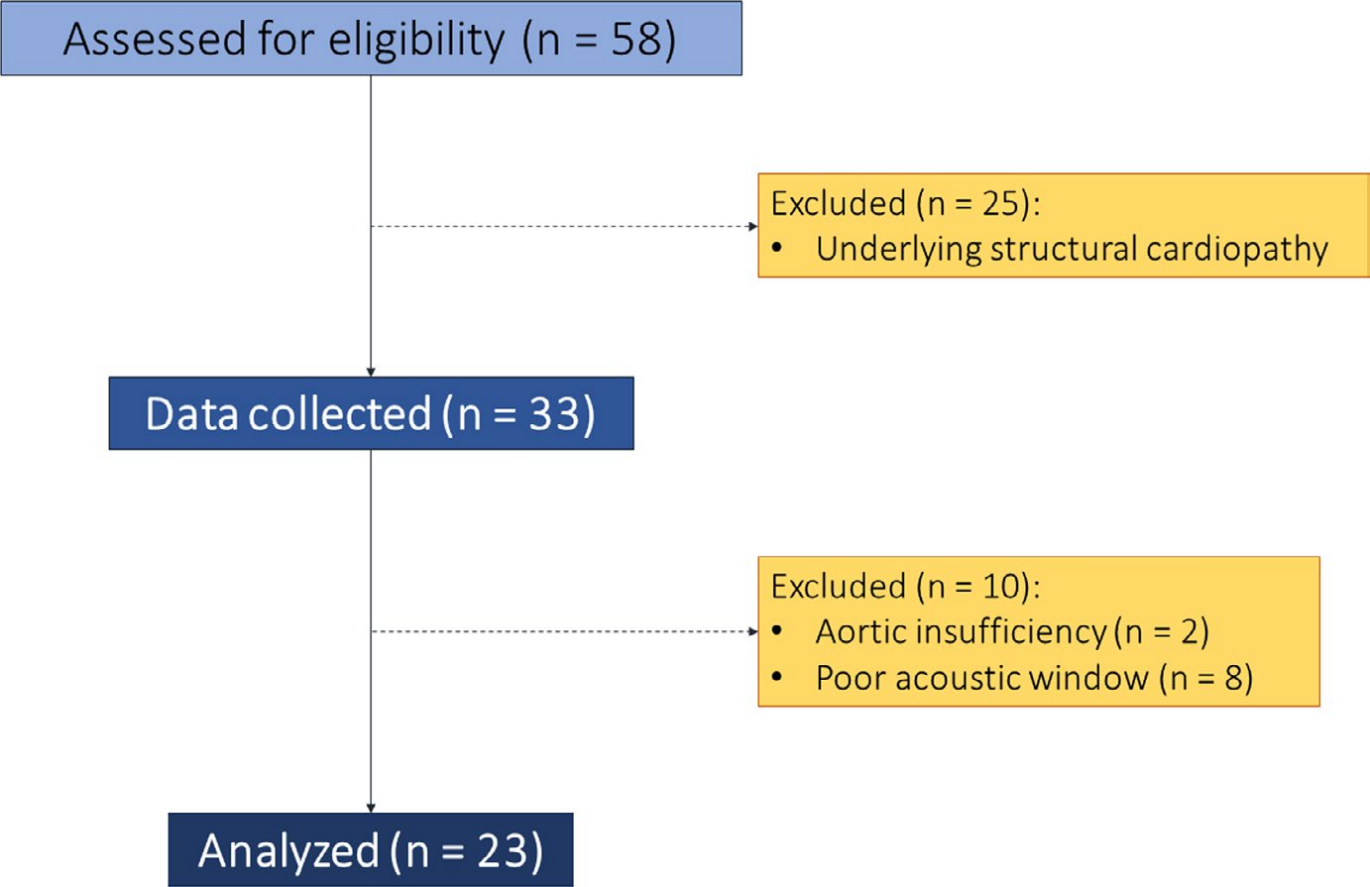
Flowchart of the patients included in the study

Twenty‐three patients were enrolled (Figure 1), aged 57 ± 17 years, 30% of which were females, 8.9 ± 6.6 days after the onset of symptoms. Only 13% suffered from Chronic Obstructive Pulmonary Disease, Global Initiative for Obstructive Lung Disease stage 1 (*n* = 2) and stage 2 (*n* = 1). Seventeen percent of patients suffered from diabetes. The anthropometric characteristics and comorbidities of the population are presented in Table 1.

**Table 1:** Anthropometrics and comorbidities of the entire population

	Population (*n* = 23)
Anthropometrics	
Age (yrs)	56 ± 17
Female (*n* ‐ %)	8 (34.7)
Weight (kg)	68.2 ± 9.5
Body Mass Index (kg/m^2^)	23.6 ± 2.7
Days from symptoms onset	8.9 ± 6.6
Comorbidities	
Chronic Obstructive Pulmonary Disease (*n* ‐ %)	3 (13)
Asthma (*n* ‐ %)	2 (8.7)
Chronic Kidney Disease (*n* ‐ %)	1 (4.3)
Diabetes	4 (17.4)

### 3.2 Gas exchange

The patients were hypoxemic (PaO_2_/FiO_2_ = 273.0 ± 102.6 mmHg) and mildly hypocapnic (PaCO_2_ = 36.2 ± 6.3 mmHg, pH = 7.45 ± 0.03) with of a moderately increased respiratory rate (20 [18–23] breaths per minute). Moreover, the A‐aO_2_ was markedly increased (115.0 [37.5–164.3] mmHg). In contrast, the CXR resulted only mildly altered (Taylor score = 2 [2–3.5] out of 5).

### 3.3 Hemodynamics

The patients had no sign of altered mental status. Body temperature was 36.5 ± 0.6°C. Only two patients had a temperature >37.5°C. They were all hemodynamically stable, with a mean systemic arterial pressure of 86.7 [80.0–88.3] mmHg (systolic 120.0 [115.0–120.0] mmHg, diastolic 70.0 [60.0–70.0] mmHg). Blood lactate levels were 1.0 [0.9–1.3] mmol/l. Heart rate was 77 ± 12 bpm and stroke index 56.5 ± 10.8 ml∙m^‐2^. Cardiac Index (CI) was on average elevated (4.32 ± 0.90 L∙min^‐1^∙m^‐2^) and 26% of the patients exhibited a CI greater than 5 L∙min^‐1^∙m^‐2^.

**Figure 2:**
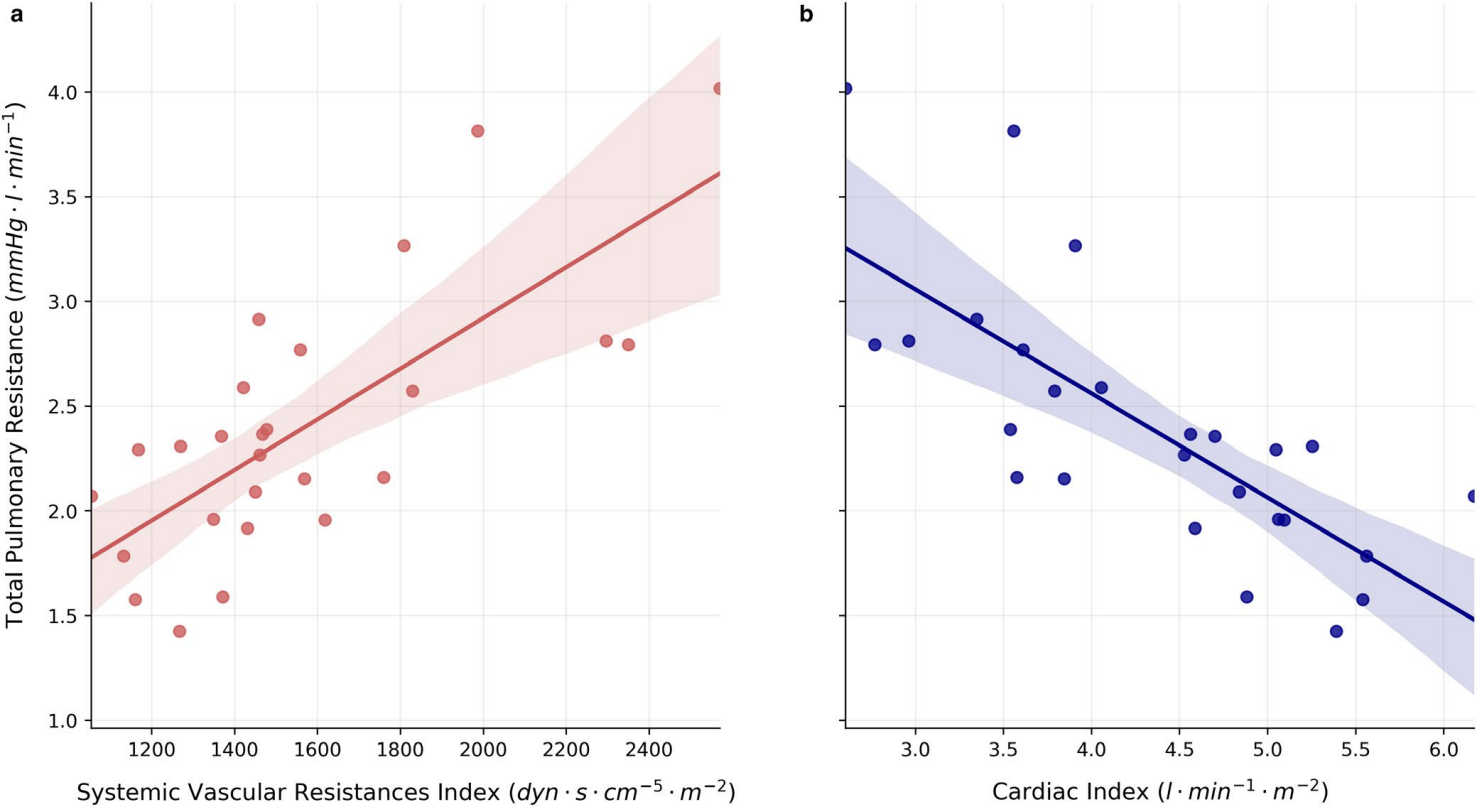
Panel a: Distribution of the systemic vascular resistance index in the whole population (physiological range 1970–2390 dyn∙s∙cm^‐5^∙m^‐2^). Panel b: Distribution of the oxygen delivery index in the whole population (physiological range 500–600 ml∙min^‐1^∙m^‐2^)

The distribution of the SVRI is reported in Figure 2, Panel A. As shown, SVRI was low in the majority of patients (1,458 [1358–1664] dyn∙s∙cm^‐5^∙m^‐2^). In Panel B we show the distribution of the Oxygen Delivery index (DO_2_i) which was elevated above the physiological value (600 ml∙min^‐1^∙m^‐2^) in 78% of the population.

In Table 2, we report the measured variables stratified above and below the median value of SVRI. Of note, in the lower SVRI group, the hypoxemia was more severe, with higher need of FiO_2_ despite an equal PaO_2_ and a remarkably higher A‐aO_2_. Despite that, DO_2_i, was particularly elevated especially in this group. Interestingly, the CXR score was similar between patients with lower or higher SVRI.

**Table 2:** Clinical characteristics of the patients stratified by the Systemic Vascular Resistance Index (SVRI) median

	Below SVRI median (*n* = 12)	Above SVRI median (*n* = 11)	*p* value
Anthropometrics			
Age (yrs)	59 ± 16	54 ± 18	.57
Body Surface Area (m^2^)	1.75 ± 0.16	1.81 ± 0.12	.35
Gas exchange			
pH	7.45 ± 0.02	7.47 ± 0.04	.11
PaO_2_ (mmHg)	78.5 [68.5 – 82.5]	80 [77.5 – 86.0]	.48
PaCO_2_ (mmHg)	36.3 ± 5.9	36.0 ± 7.0	.90
FiO_2_	0.38 [0.21 – 0.60]	0.21 [0.21 – 0.34]	.06
SaO_2_ (%)	98 ± 2	97 ± 2	.13
PaO_2_/FiO_2_ (mmHg)	233.0 ± 108.0	316.6 ± 79.4	.046*
A‐aO_2_ (mmHg)	168 ± 120	73.05 ± 52	.024*
Respiratory rate (bpm)	22.8 ± 5.5	19.4 ± 3.2	.072
Severity Chest X‐Ray	2.0 [2.0 – 3.25]	2.0 [1.5 – 3.5]	.74
Hemodynamics			
Body temperature (°C)	36.5 ± 0.7	36.5 ± 0.6	.72
Heart rate (bpm)	83 ± 7	71 ± 13	.012*
Systolic arterial pressure (mmHg)	120 [115–125]	120 [118–120]	.94
Mean arterial pressure (mmHg)	82.8 ± 7.4	84.6 ± 8.3	.59
Dyastolic arterial pressure (mmHg)	65.4 ± 7.2	68.2 ± 10.6	.48
Left ventricle ejection fraction (%)	60 [57–66]	62 [61–64]	.82
Tricuspid Annular Plane Systolic Excursion (mm)	21 [20–22]	21 [21 –22]	.97
Systolic pulmonary pressure (mmHg)	26.3 ± 5.3	26.2 ± 4.3	.98
Mean pulmonary pressure (mmHg)	18.0 ± 3.2	18.0 ± 2.6	.98
Estimated right atrial pressure (mmHg)	3 [3–4]	3 [3–5]	.38
Left Ventricle Outflow Tract (mm)	23.1 ± 1.6	22.0 ± 1.6	.13
Stroke index (ml∙m^−2^)	59.2 ± 7.6	53.6 ± 13.1	.24
Cardiac index (l∙min^−1∙^m^−2^)	4.92 ± 0.73	3.68 ± 0.58	<.001*
Systemic vascular resistance index (dyn∙s∙cm^−5^∙m^−2^)	1,302 ± 141	1798 ± 363	<.001*
Oxygen delivery index (ml∙min^−1^∙m^−2^)	859.4 ± 217.7	642.9 ± 129.4	.009*
Total Pulmonary Resistance (mmHg∙l∙min^−1^)	2.08 [1.88–2.32]	2.57 [2.32–3.04]	.010*
Laboratory			
Hemoglobin (g/dl)	12.6 ± 2.4	12.8 ± 1.7	.80
White blood count (10^9^ cells/L)	6.87 ± 1.95	4.81 ± 1.26	.006*
Lymphocytes (10^9^ cells/L)	1.1 [0.9–1.7]	1.3 [1.0–1.7]	.56
Monocytes (10^9^ cells/L)	0.35 ± 0.14	0.46 ± 0.25	.20
D‐dimers (ng/ml)	396.5 [291–1026]	437 [336–1037]	1.0
Fibrinogen (mg/dl)	619 ± 128	607 ± 130	.83
International Normalized Ratio	1.28 ± 0.14	1.23 ± 0.10	.37
Creatinine (mg/dl)	0.78 [0.70–0.92]	0.76 [0.69–0.81]	.33

^*^ p < .05.

**Figure 3:**
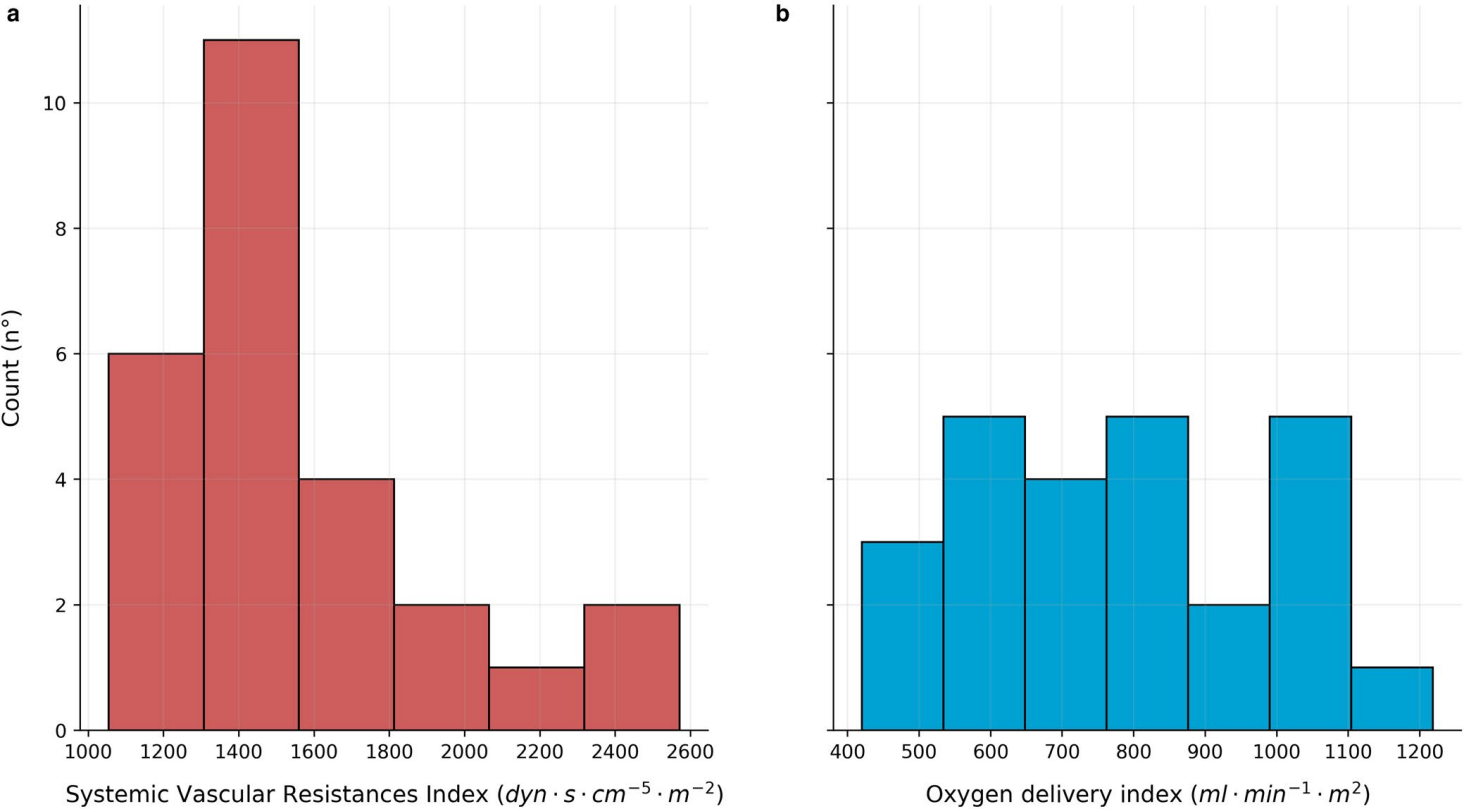
Panel a: relationship between the Total Pulmonary Resistance and the Systemic Vascular Resistance index (*p* < .001, R^2^ = 0.55). Panel b: relationship between the Total Pulmonary Resistance and the Cardiac index (*p* < .001, R^2^ = 0.56)

No intracardiac shunts, moderate‐to‐severe TR or additional echocardiographic signs suggesting pulmonary hypertension (PH) were detected. Right heart function, dimensions, and pressures were within physiological limits: Tricuspidal Annular Plane Systolic Excursion was 21 [20–22] mm, PAPs 26.2 ± 4.7 mmHg, PAPm 18.0 ± 2.9 mmHg, estimated RAP 3.0 [3.0–4.5] mmHg. Interestingly, the TPR was normal (2.3 [2.1–2.7] mmHg∙L^‐1^∙min^‐1^). In Figure 3, Panel A, we report the relationship between TPR and SVRI. As shown, the two variables were remarkably proportional (*p* < .001, R^2^ = 0.55). In contrast, TPR and CI were inversely related (*p* < .001, R^2^ = 0.56).

## 4 DISCUSSION

In this study, we found that in the early stages of the disease, COVID‐19 infection leads to a hyperdynamic circulatory state characterized by an elevated CI and low SVRI. Moreover, patients with particularly low SVRI, despite a supranormal DO_2_i, had more severe oxygenation impairment. The right heart function was unaffected, and pulmonary pressures were normal, despite the high CI, particularly in the low SVRI range. The negative Quick SOFA score (no altered mentation, respiratory rate <22 bpm, systolic arterial blood pressure >100 mmHg), the absence of fever, normal white blood cell count, and lactates within normal ranges make the hypothesis of a septic status unlikely.

### 4.1 Systemic hemodynamics

The effects of hypoxemia on cardiac output (Kontos et al., 1967) and on the SVRI is known for decades (Guyton et al., 1964). Indeed, to increase the oxygen delivery, heart rate and stroke volume increase and the arterial smooth muscles relax, thereby lowering the peripheral resistances. However, most of the observations show that this homeostatic response actually occurs when PaO_2_ and, consequently, SaO_2_ decrease to such extent that the arterial oxygen content (CaO_2_) is extensively reduced (Fernandes et al., 2018). Our population, instead, thanks to the oxygen administration, showed a near normal PaO_2_ and SaO_2_. This, together with the increased cardiac output, led to a supranormal DO_2_i, particularly in the low SVRI group. Therefore, it is tempting to speculate that hypoxemia alone may not fully justify the peripheral vasodilation and a direct action of the virus, given also its tropism for the ACE‐2 receptor, may be at play., Viral sepsis has been suggested in COVID‐19, but such an entity is stilly poorly characterized (Li et al., 2020). Our findings may represent the early hyperdynamic phase of viral sepsis. Furthermore, Sonzogni et al. (2020) described severe alterations in hepatic blood flow with massive portal vessel dilations in autopsies from COVID‐19 patients. Peripheral shunting and the associated reduction in transit time may per se justify the high CI and low SVRI observed in our population.

In this context, it is possible that the overall reduction of vasomotor tone further worsens the hypoxemia. Notably, patients with lower SVRI were more hypoxic, despite similar severity of the chest x‐ray infiltrates. The hyperdynamic state may itself lead to further V_A_/Q mismatch, through a reduction of the transit time and diffusion limitation as during exercise (Dempsey & Wagner, 1999). A high CI itself leads to a global lowering of the V_A_/Q ratio. For this reason, the beneficial effects of Continuous Positive Airway Pressure (CPAP) on oxygenation may act, at least in part, by reducing the disproportionate CO. Lung recruitability in COVID‐19 is moderately low (Pan et al., 2020) and blood diversion with cardiac output reduction may justify the reported oxygenation improvement at high Positive End Expiratory Pressure (PEEP).

### 4.2 Pulmonary hemodynamics

Reportedly, pulmonary embolism and microthrombosis are distinguishing features of COVID‐19 (Wichmann et al., 2020) and our non‐elderly cohort showed elevated D‐dimers (max 70,230 ng/ml). Thus, we would have expected to find signs of increased pulmonary pressure, but this was not the case. Indeed, we found normal estimated pulmonary pressures and TPR were lower in patients with higher CI, suggesting that pulmonary capillary recruitment worked efficiently. We are by no mean implying that pulmonary microembolism is not present in the early stages of the disease. Rather, our findings suggest that the balance between vasodilation and vessel occlusion, at this stage, is shifted toward the former. A recent interesting report showed increased intrapulmonary neoangiogenesis (Ackermann et al., 2020) that, by increasing the pulmonary vascular bed may, at least partly, explain this finding. Pathological vasodilation of the pulmonary vasculature is also supported by the short duration and quick reversibility of the beneficial oxygenation effect during prone position (Elharrar et al., 2020) and the positive response to almitrine infusion (Losser et al., 2020).

## 5 LIMITATIONS

These preliminary data have several limitations, primarily the low number of patients enrolled and the indirect estimation of the echo‐acquired pulmonary pressures. Even if we excluded subjects with probable pathological elevation of wedge pressure, these values must be confirmed by pulmonary artery catheterization. It is worth noting, however, that positive pressure ventilation and/or development of overt pulmonary embolism may change dramatically the overall picture.

## 6 CONCLUSIONS

In the early stages of COVID‐19 infection, we detected a hyperdynamic state characterized by high CI and low SVRI. This was in turn associated with the severity of the hypoxemia, in absence of macrohemodynamic signs of pulmonary embolism. Rather, pulmonary pressures were normal despite the high CI, favoring the hypothesis of a reduction of the pulmonary vasomotor tone or an increased vascular bed.

## CONFLICT OF INTERESTS

The authors report no conflicts of interest to disclose.

## AUTHOR CONTRIBUTION

MS, AL, GBF, EC, CBC, and AG collected the data. MB analyzed the data. MB, MS, and AG wrote the first draft of the manuscript. All authors critically revised and approved the manuscript.

## ETHICAL STATEMENT

The study was approved by the ethical committee of the institution.

## Data Availability

The dataset of the study is available upon a justified request.
